# Effects of ischemic pre- and postconditioning on HIF-1α, VEGF and TGF-β expression after warm ischemia and reperfusion in the rat liver

**DOI:** 10.1186/1476-5926-10-3

**Published:** 2011-07-19

**Authors:** Anders R Knudsen, Anne-Sofie Kannerup, Henning Grønbæk, Kasper J Andersen, Peter Funch-Jensen, Jan Frystyk, Allan Flyvbjerg, Frank V Mortensen

**Affiliations:** 1 Department of Surgical Gastroenterology L, Aarhus University Hospital, Aarhus, Denmark; 2 The Medical Research Laboratories, Clinical Institute, Aarhus University Hospital, Aarhus, Denmark; 3 Department of Medicine V, Aarhus University Hospital, Aarhus, Denmark

## Abstract

**Background:**

Ischemic pre- and postconditioning protects the liver against ischemia/reperfusion injuries. The aim of the present study was to examine how ischemic pre- and postconditioning affects gene expression of hypoxia inducible factor 1α (HIF-1α), vascular endothelial growth factor A (VEGF-A) and transforming growth factor β (TGF-β) in liver tissue.

**Methods:**

28 rats were randomized into five groups: control; ischemia/reperfusion; ischemic preconditioning (IPC); ischemic postconditioning (IPO); combined IPC and IPO. IPC consisted of 10 min of ischemia and 10 min of reperfusion. IPO consisted of three cycles of 30 sec. reperfusion and 30 sec. of ischemia.

**Results:**

HIF-1α mRNA expression was significantly increased after liver ischemia compared to controls (p = 0.010). HIF-1α mRNA expression was significantly lower in groups subjected to IPC or combined IPC and IPO when compared to the ischemia/reperfusion group (p = 0.002). VEGF-A mRNA expression increased in the ischemia/reperfusion or combined IPC and IPO groups when compared to the control group (p < 0.05).

**Conclusion:**

Ischemic conditioning seems to prevent HIF-1α mRNA induction in the rat liver after ischemia and reperfusion. This suggests that the protective effects of ischemic conditioning do not involve the HIF-1 system. On the other hand, the magnitude of the HIF-1α response might be a marker for the degree of I/R injuries after liver ischemia. Further studies are needed to clarify this issue.

## Background

Colorectal cancer is a leading form of cancer in the Western world. Approximately 50% of patients with this disease have, or will eventually develop, liver metastases. Surgical removal of those metastases remains the treatment of choice, with a five year survival rate of 37%-58% after resection [1-3]. Major hemorrhage and blood transfusion during liver resection is related to an increase in morbidity and mortality [4-6]. Vascular clamping is a frequently used method for reducing blood loss [7]. Several studies have shown that the normal livers tolerate periods of continuous warm ischemia up to 90 min and intermittent warm ischemia up to 120 min [8-10].

However, ischemia/reperfusion (I/R) injury of the liver is an unfortunate side effect of this method, ranging from slightly elevated liver enzymes to acute liver failure [11]. Ischemic pre- or postconditioning (IPC or IPO), defined as brief periods of ischemia and reperfusion before or after sustained ischemia, have proven to increase the ability of organs to tolerate I/R injury [12-16]. The precise mechanisms responsible for the hepatoprotection from ischemic injuries are only partially known. Focus has been on a system of hypoxia inducible factors (HIF), where especially HIF-1 appears to have a major role in cellular adaptation to hypoxia. HIF-1 mediates essential homeostatic responses to cellular hypoxia by up-regulating gene transcription, via specific DNA motif called hypoxia response elements, and activating target genes. HIF-1 is a heterodimer protein consisting of an α and β-subunit. The β-subunit is expressed ubiquitously in most cells, whereas expression of the α-subunit is controlled by cellular oxygen tension. Under normal conditions the HIF-1α protein is degraded via an oxygen dependent system. By contrast, hypoxia inactivates the degradation causing stabilization of the HIF-1α protein, which then translocate to the nucleus and forms dimers with the β-subunit [17]. The active form of HIF-1 transactivates other genes as vascular endothelial growth factor (VEGF) and transforming growth factor β1 (TGF-β1) [18,19]. VEGF is an important growth factor involved in angiogenesis. It is a multifunctional protein, with several effects on endothelial cells to promote the formation of new vessels. Furthermore, it stimulates the production of hepatocyte growth factor (HGF), which is regarded as an initiator of liver regeneration [20]. TGF-β1 is a member of the superfamily of cytokines. In the liver, TGF-β1 has anti-inflammatory properties and stimulates cell proliferation as well as differentiation [20].

Besides I/R injuries, another possible drawback of liver ischemia in cancer surgery could be growth stimulation of micrometastases. Several studies indicate that the outgrowth of micrometastases is stimulated by I/R injuries during hepatic resections [21-23]. Outgrowth of these micro metastases may at least in part, be stimulated by an increased HIF-1α stabilization [22]. As mentioned above, HIF-1α activates other genes such as VEGF and TGF-β. Especially VEGF is an important growth factor involved in angiogenesis [24-26]. In this sense a stimulation of HIF-1α, via liver ischemia, could be a double-edged sword; *i.e*., it protects the liver against I/R injuries, but a side effect could be the growth stimulation of micrometastases through angiogenesis.

The aim of the present study was to examine how ischemia, with or without IPC and IPO, affects the expression of HIF-1α and the target genes VEGF and TGF-β1, in rodent liver.

## Methods

**Figure 1:**
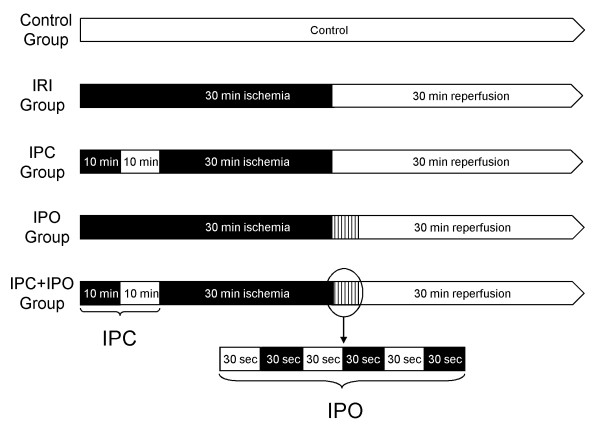
**Experimental protocol of the five groups**. Black areas represent periods of hepatic ischemia; white areas represent periods of normal hepatic blood perfusion. Liver biopsies were collected at the end of each experiment. CG, Control group. IRI, 30 min of ischemia. IPC, ischemic preconditioning + 30 min of ischemia. IPO, 30 min ischemia + ischemic postconditioning. IPC+IPO, ischemic preconditioning + 30 min of ischemia + ischemic postconditioning.

The surgical and experimental protocols were approved by the Danish Animal Research Committee, Copenhagen, Denmark according to license number 2007/561-1311 and followed the *Guide for the Care and Use of Laboratory Animals *published by the National Institute of Health. Twenty-eight adult male Wistar rats weighing 300-350 g (M&B Taconic, Eiby, Denmark) were used for the experiment. Animals were housed in standard animal laboratories with a temperature maintained at 23°C and an artificial 12-hour light-dark cycle, with food and water *ad libitum*, until the time of the experiment. The rats were randomly divided into five groups as follows: sham operated control (CG) (*n *= 4); pure ischemia and reperfusion (IRI) (*n = 6*); IPC (*n = 6*); IPO (*n = 6*); and IPC+IPO (*n = 6*) (Figure 1). All animals were anaesthetized with 0.75 ml/kg Hypnorm s.c. (Fentanyl/Fluanisone, Jansen Pharma, Birkerød, Denmark) and 4 mg/kg Midazolam s.c. (Dormicum, La Roche, Basel, Switzerland) and placed on a heated pad. A midline laparotomy was performed and total hepatic ischemia was accomplished using a microvascular clamp placed on the hepatoduodenal ligament, *i.e*., performing the Pringle maneuver. Reflow was initiated by removal of the clamp. Discoloration of the liver was used as a positive marker for hepatic ischemia. Reperfusion was ascertained by the return of the normal brown/reddish color of the liver. The experimental protocol was performed as described in Figure 1. At the end of each experiment after 30 min of reperfusion, a biopsy was taken from the right liver lobe, immediately frozen in liquid nitrogen and stored at -80°C for further analysis. Blood samples were collected from the common iliac artery in tubes for measurement of alanine aminotransferase (ALAT), alkaline phosphates and bilirubin, and analyzed immediately hereafter. All rats were subsequently killed with an overdose of pentobarbital.

### Quantitative Real-Time PCR (RT-PCR)

After homogenization of liver tissue by the use of a MM301 Mixer Mill (Retsch, Haan, Germany), total cellular RNA was extracted from the liver tissue using a 6100 Nucleic Acid PrepStation (Applied Biosystems, Foster City, CA, USA). The quality of rRNA was estimated by agarose gel electrophoresis by the appearance of two distinct bands visible by fluorescence of ethide bromide representing intact rRNA. The amounts of RNA extracted were quantified by measuring the absorbance by spectrophotometry, at 260 nm. Reverse transcription from RNA to DNA was performed with a Multiscribe Reverse Transcriptase kit from Applied Biosystem at 25°C for 10 min, at 48°C for 30 min and at 94°C for 29 sec. The PCR was performed in triplicates of each sample in a volume of 25 μL in each well containing RNA, TaqMan Universal PCR MasterMix and a primer of the target, *i.e*., HIF-1α (Rn00577560_m1), TGF-β (Rn00572010_m1) and VEGF-A (Rn4331348), and a primer of the housekeeping gene, 18S (4319413), all purchased from Applied Biosystems. Each RT-PCR reaction ran at 50°C for 2 min, at 95°C for 10 min and in 40 cycles changing between 95°C for 15 sec. and 60°C for 1.30 min [27].

### PCR Data analysis

Data was analyzed with the ABI Prism 7000 Sequence Detector Software from Applied Biosystems. The output of amplification was measured in the exponential phase of the reaction as the threshold cycle/Ct-value, which is defined as the cycle number at which amplification products are detected corresponding to the point where fluorescent intensity exceeds the background fluorescent intensity, which is 10 × the standard deviation of the baseline. The average of triplicates from each sample was used. The relative quantification of target gene was calculated using the formula: (1/2)^Ct-target gene- Ct-housekeeping gene^, which is described in the Users Bulletin 2, 1997 from Perkin-Elmer (Perkin-Elmer Cetus, Norwalk, CT, USA) [27].

### Statistical analysis

Statistical analysis were performed by SPSS^® ^11.0 programs (SPSS Inc., Chicago, Illinois, USA). All data is expressed as mean ± SEM. Comparisons of data between groups were performed by non-parametric Kruskal-Wallis (ANOVA) test followed by the Mann-Whitney *U*-test. A p value < 0.05 was considered significant.

## Results

### Liver parameters

**Figure 2:**
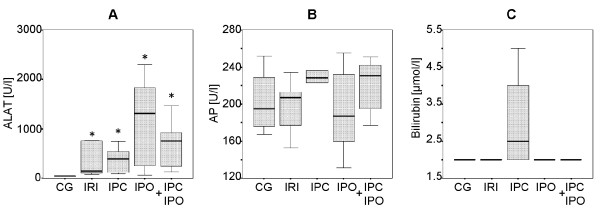
**Blood samples including ALAT (A), alkaline phosphatase (AP) (B) and bilirubin (C) levels**. Samples 30 min after reperfusion in CG, Control group. IRI, 30 min of ischemia. IPC, ischemic preconditioning + 30 min of ischemia. IPO, 30 min ischemia + ischemic postconditioning. IPC+IPO, ischemic preconditioning + 30 min of ischemia + ischemic postconditioning. * indicates p ≤ 0.01 compared to the control group.

Blood samples showed a significant increase in ALAT in group IRI (334 ± 135 U/L), IPC (377 ± 104 U/L), IPO (1177 ± 379 U/L) and IPC+IPO (710 ± 199 U/L) compared to the control group (40 ± 2 U/L) (CG vs. IRI, IPC, IPO, and IPC+IPO, p = 0.01). No significant differences were found in ALAT between groups IRI, IPC, IPO and IPC+IPO. Alkaline phosphates and bilirubin were comparable between groups (Figure 2).

### HIF-1α expression

**Figure 3:**
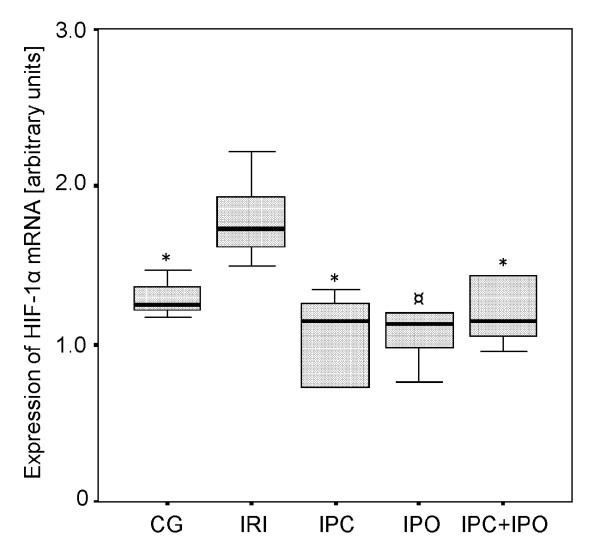
**Expression of HIF-1α mRNA**. Expression after 30 min of reperfusion. CG, Control group. IRI, 30 min of ischemia. IPC, IPC + 30 min of ischemia. IPO, 30 min ischemia + IPO. IPC+IPO, IPC + 30 min of ischemia + IPO. * indicates p ≤ 0.01 compared to group IRI. ¤ indicates p = 0.065 compared to group IRI.

In the IRI group the expression of HIF-1α mRNA was significantly increased after 30 min of reperfusion compared to the control group (p ≤ 0.01). In the IPC group HIF-1α mRNA expression was significantly lower than the IRI group (p ≤ 0.01). In rats subjected to IPO there was a tendency towards lower HIF-1α mRNA expression compared to the IRI group (p = 0.065). In the IPC+IPO group HIF-1α mRNA expression was significantly lower compared to the IRI group (IRI vs. IPC+IPO, p ≤ 0.01). The HIF-1α mRNA levels were comparable between group CG, IPC, IPO and IPC+IPO (Figure 3)

### VEGF expression

**Figure 4:**
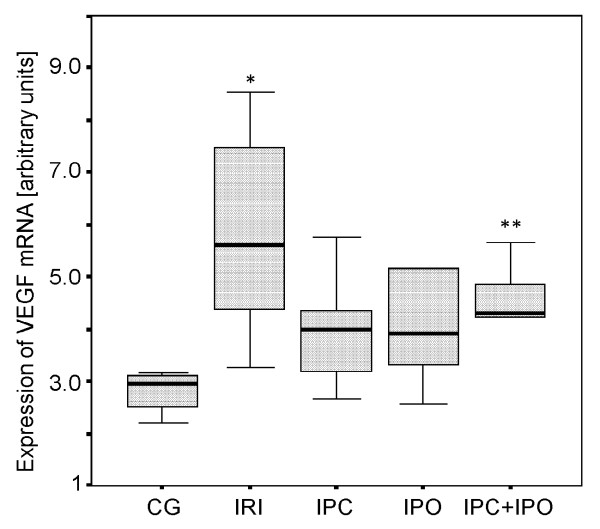
**Expression of VEGF mRNA**. Expression after 30 min of reperfusion. CG, Control group. IRI, 30 min of ischemia. IPC, IPC + 30 min of ischemia. IPO, 30 min ischemia + IPO. IPC+IPO, IPC + 30 min of ischemia + IPO. *indicates p ≤ 0.01 compared to group CG. **indicates p ≤ 0.038 compared to group CG.

As shown in Figure 4, VEGF mRNA expression was significantly increased in the IRI group compared to the control group (p ≤ 0.01). When applying IPC+IPO VEGF mRNA expression was also increased compared to the control group (p ≤ 0.038). No significant differences were observed between groups IPC, IPO and the control group (IPC vs. CG, p ≤ 0.067) and (IPO vs. CG, p ≤ 0.067).

### TGF-β1 expression

**Figure 5:**
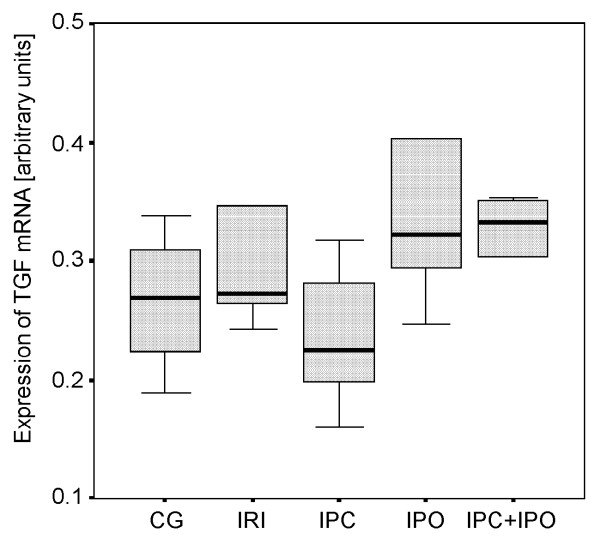
**Expression of TGF-β1 mRNA**. Expression after 30 min of reperfusion. CG, Control group. IRI, 30 min of ischemia. IPC, IPC + 30 min of ischemia. IPO, 30 min ischemia + IPO. IPC+IPO, IPC + 30 min of ischemia + IPO.

No differences in TGF-β1 mRNA expression were observed between the five groups (Figure 5).

## Discussion

As expected HIF-1α mRNA expression was increased significantly in rats subjected to 30 minutes of warm liver ischemia and 30 minutes of reperfusion compared to the control group. The main finding of this study was an absent of HIF-1α induction in IPC or IPC+IPO treated animals. In both of these groups, the expression levels were similar to that of CG. In the IPO group the same tendency towards an absent induction of HIF-1α was observed although not significant. VEGF mRNA expression increased significantly when applying 30 min of ischemia without ischemic conditioning compared to sham operated controls. IPC+IPO also showed increased VEGF mRNA expression compared to sham operated controls, whereas neither ischemia nor ischemic conditioning affected hepatic TGF-β expression.

The cytoprotective effects of IPC, defined as brief periods of ischemia and reperfusion prior to prolonged ischemia, on I/R injuries to the liver have become indisputable with an increasing number of studies supporting this fact [12-14]. The IPC protocol used in this study has previously been shown to induce hepatoprotection against I/R injuries. We choose circulating ALAT as marker of hepacellular injuries, as this parameter is well established and known to correlate to the degree of injury [28-30]. However, we were unable to see any hepatoprotective effects as assessed by changes in liver parameters. In previous studies with the same IPC protocol, longer periods of ischemia and longer reperfusion periods were utilized [12,14,31]. This might explain why we were not able to demonstrate protective effects of IPC and IPO as judged by liver parameters, *i.e*., the duration of ischemia was too short. Furthermore, 30 min of reperfusion might be too short follow up to demonstrate the full extent of the I/R injuries. The cytoprotective effect of IPO, defined as brief periods of ischemia and reperfusion after liver ischemia, is less well established [15,16]. In the present study, we could not demonstrate any hepatoprotective effects of IPO assessed by liver parameters, and we speculate that the explanation may be the same as above. We choose the actual time protocol with 30 minutes of ischemia because we wanted to create a setting relevant for normal clinics. Even though longer periods of liver ischemia have been safely applied, most surgeons would be reluctant to induce more than 30 minutes of ischemia on the liver.

The mechanisms responsible for the protective effects of IPC and IPO are only partially understood. In the present study, IPC resulted in a significantly lower expression of HIF-1α mRNA compared with rats subjected to liver ischemia without IPC. This leads us to conclude that HIF-1α, in our model of modest I/R-injuries, does not seem to be a mediator of the cyto-protective effects of IPC. In rats subjected to IPO there was a tendency towards lower HIF-1α mRNA expression, although not significant, when compared to the sheer liver ischemia group. This indicates that HIF 1α is not involved in the cytoprotective effects of IPO. In this sense, the HIF-1α mRNA response could to be a marker of the degree of I/R injury, *i.e*., the higher HIF-1α mRNA response after ischemia, the more pronounced I/R injuries. Further studies need to be performed to address this issue, but it is first and foremost supported in a study by Cursio et al., where they showed that the expression of HIF-1 and the degree of apoptosis was increased in rats subjected to 120 min of warm liver ischemia compared to non-ischemia [32]. Another study supporting the conclusion in the present paper is that by Feinman et al. [33]. They used partially HIF-1 deficient mice in a hemorrhagic shock model and concluded that HIF-1 activation was necessary for ischemic gut mucosal injury.

The expression of VEGF mRNA was regulated upwards by the ischemic episodes in the group subjected to sustained ischemia and in the IPC+IPO group. A higher expression of VEGF in the group with liver ischemia only, correlates with the elevated HIF-1α expression in this group. TGF-β expression levels were not affected in any of the groups. Both VEGF and TGF-β are, as previously described, genes that are regulated downstream of HIF-1α. However, as this study only focuses on the expression levels after 30 min of reperfusion, we cannot be sure that we are measuring the full effect of the changed HIF-1α levels. If we had followed the expression levels over time, we might have seen a more direct correlation, as already reported [34].

## Conclusions

Ischemic conditioning seems to prevent HIF-1α mRNA induction in the rat liver after ischemia and reperfusion. This suggests that the protective effects of ischemic conditioning do not involve the HIF-1 system. On the other hand, the magnitude of the HIF-1α response might be a marker for the degree of I/R injuries after liver ischemia. Further studies need to be performed to elucidate this matter.

## Competing interests

The authors declare that they have no competing interests.

## Authors' contributions

Study conception and design: ARK, A-SK, FVM. Acquisition of data: ARK, A-SK, KJA. Analysis and interpretation of data: ARK, A-SK, HG, KJA, PF-J, JF, AF, FVM. Drafting of manuscript: ARK, A-SK, KJA, FVM. Critical revision of manuscript: ARK, A-SK, HG, KJA, PF-J, JF, AF, FVM. All authors read and were in accordance with the final manuscript.
